# Occupational exposure to blood, hepatitis B vaccine knowledge and uptake among medical students in Cameroon

**DOI:** 10.1186/1472-6920-13-148

**Published:** 2013-11-08

**Authors:** Jean Jacques N Noubiap, Jobert Richie N Nansseu, Karen K Kengne, Shalom Tchokfe Ndoula, Lucy A Agyingi

**Affiliations:** 1 Faculty of Medicine and Biomedical Sciences, University of Yaoundé I, Yaoundé, Cameroon; 2 Internal Medicine Unit, Edéa Regional Hospital, PO Box 100, Edéa, Cameroon; 3 Mother and Child Centre, Chantal Biya Foundation, Yaoundé, Cameroon; 4 Guidiguis Health District, Guidiguis, Cameroon; 5 Faculty of Science, University of Dschang, Dschang, Cameroon

**Keywords:** Hepatitis B vaccine, Accidental exposure to blood, Medical students, Cameroon

## Abstract

**Background:**

Hepatitis B virus (HBV) is the most contagious blood borne pathogen. The risk of occupational exposure to HBV among health care workers is a major concern, especially medical trainees. In this study we describe the knowledge of risk factors for HBV infection, history of accidental exposure to blood, awareness of HBV vaccine and the vaccination status among medical students in Cameroon.

**Methods:**

In April 2012, a cross-sectional survey was carried out using a pretested self-administered questionnaire among 111 medical students.

**Results:**

Sixty-two students (55.9%) had had at least one accidental exposure to blood since the beginning of their medical training, with a median of 2 (IQR, 1-3) exposures. There was a good knowledge of the risk factors for HBV infection and awareness of HBV vaccine among participants. However, only 20 (18%) participants had completed the three doses of primary HBV vaccination. Furthermore, only 2 of the 20 (10%) adequately vaccinated participants had a post-vaccination test to confirm a good immune response and thus an effective protection against HBV infection. The main reason for not being vaccinated was lack of money to pay for the vaccine (45.6%). Forty seven (42.3%) participants had been sensitized by their training institutions about the importance of HBV vaccination. These were more likely to be vaccinated compared to those who had not been sensitized (p<0,001).

**Conclusion:**

There is a high rate of accidental exposure to blood and a very low HBV vaccination uptake in medical students in Cameroon, leading to a high occupational risk of HBV infection. HBV vaccination should be strongly recommended for medical students and the vaccine made available free of charge at the beginning of their training.

## Background

It is estimated that more than two billion people are infected with HBV worldwide and about 350 million of them suffer from chronic HBV infection; mainly liver cirrhosis and hepatocellular carcinoma [1]. The prevalence of HBV chronic infection is particularly high in sub-Saharan Africa, ranging from 7 to 26% [2]. Health care workers are at a high risk of HBV infection through occupational exposure to blood, and the incidence of this infection among them has been estimated to be 2-4 times the level in the general population [3]. As part of occupational safety measures, all health care workers are required to be vaccinated against HBV [4,5]. Unfortunately, the World Health Organization has estimated that HBV vaccination coverage amongst health care workers is only 18-39% in low and middle-income countries compared to 67-79% in high-income countries [6].

HBV vaccination is now part of the national routine immunization program for children in Cameroon. HBV vaccination in health care workers and trainees in Cameroon is recommended but not strictly enforced. Health care workers have been reported to have the highest occupational risk of HBV infection during their health professional training [6]. To the best of our knowledge, there is no published information on HBV vaccination uptake among medical students in Cameroon.

This study sought to determine, among medical students in Cameroon, their knowledge of risk factors for HBV infection, history of accidental exposure to blood, awareness of HBV vaccine and their vaccination status.

## Methods

### Ethical considerations

This study was performed in accordance with the guidelines of the Helsinki Declaration and was approved by the Institutional Ethical Review Board of the Faculty of Medicine and Biomedical Sciences of Yaoundé. Written informed consent was obtained from all the participants.

### Study design and setting

This is an observational cross-sectional study conducted in April 2012 at the Faculty of Medicine and Biomedical Sciences of the University of Yaoundé I, Cameroon. This faculty is the oldest and the greatest among the four public faculties of medicine in Cameroon.

### Study participants and sampling

To be eligible students had to be of the clinical years (years IV, V and VI), consenting and willing to fill a self-administered questionnaire. Participants were recruited through a non-randomized, simple and consecutive sampling of eligible medical students met either at the campus of the faculty or the teaching hospitals during the period of the study.

### Data collection, variables and measurements

For the collection of data, we used a structured pretested questionnaire that was prepared based on some previous studies [7,8], and considered by a panel of consulting experts. The questionnaire’s validity and reliability were confirmed by Cronbach’s alpha coefficient (alpha = 0.72). The questionnaire had four sections: demographic and academic characteristics, knowledge of the risk factors for HBV infection and HBV vaccine, history of accidental exposure to blood, and a section on perception of HBV vaccine and vaccination status. The questionnaire was self-administered: consenting participants were given printed copies of the questionnaire and allowed time to fill them at their will and convenience. Participants then returned these questionnaires anonymously to the researchers.

In the context of the present study, we considered participants adequately vaccinated if they had received a minimum of three intramuscular injections of 20 micrograms of HBsAg (hepatitis B surface antigen) at a schedule of 0, 1 and 6 months; thus completing the minimum primary HBV vaccination series. Participants were considered inadequately vaccinated if they had started HBV vaccination but did not complete the three doses of primary vaccination, and not vaccinated if they had never received a dose of an HBV vaccine. For the evaluation of the general knowledge of the risk factors for HBV infection and HBV vaccine, we calculate the mean percentage of correct answers for all the questions on the risk factors and the HBV vaccine. Their knowledge was considered “good” if the mean percentage of correct answers was equal or greater than 75%, “fair” if it was less than 75% and equal or greater than 50%, and “poor” if it was less than 50%.

### Data analysis

Data was coded, entered and analysed using the Statistical Package for Social Science (SPSS) version 20.0 for Windows (SPSS, Chicago, Illinois, USA). We described continuous variables using either medians with interquartile ranges (IQR) or means with standard deviations, and categorical variables using their frequencies and percentages. The Chi-square test or its equivalents were used to compare qualitative variables and a p-value less than 0.05 was considered statistically significant.

This manuscript was written following STROBE guidelines for the reporting of observational studies [9].

## Results

In this study we enrolled 111 medical students in clinical years (years IV, V and VI) at the medical school during the 2011-2012 academic year. Their ages ranged from 20 to 27 years with a mean age of 23.04 ± 1.1 years and 51.3% (57/111) were male. Eighteen (16.2%) were year IV, 48 (43.2%) year V and 45 (40.5%) year VI medical students.

### History of accidental exposure to blood

From the beginning of their medical training, 62 (55.9%) had had at least one accidental exposure to blood, with a median of 2 (IQR, 1-3) exposures. Up to 21.7% (33/152) of the exposures had occurred within the preclinical years (years I, II and III) during non-academic clinical training courses, and 59.2% (90/152) had occurred during the first and second clinical years (years IV and V). Among the 62 students who had had at least one accidental exposure to blood, only 26 (42%) had always notified their exposure and 39 (62.9%) never considered the risk of HBV infection after exposure but only the risk of HIV infection.

### Knowledge of the risk factors for HBV infection

As depicted in Table 1, there was a good knowledge of the risk factors for HBV infection among participants (83.2% of correct answers). Eighty-seven (78.4%) thought that they were at a greater risk of becoming infected with HBV than the general population.

**Table 1:** Knowledge of risk factors and HBV vaccination among 111 medical students in Cameroon

**Statements**	**Correct answers N (%)**
1. HBV is the most contagious blood-borne pathogen through accidental exposure to blood	60 (54.1)
2. Contact of healthy skin with infected blood is a risk factor of HBV infection	103 (92.8)
3. Injury with needle contaminated with infected blood is a risk factor of HBV infection	107 (96.4)
4. Contact of abraded skin with infected body fluid is a risk factor of HBV infection	101 (91)
5. Contact of eyes with infected blood is a risk factor of HBV infection	81 (73)
6. HBV could be transmitted through sexual intercourse	98 (88)
7. HBV could be transmitted through oro-fecal route	81 (73)
8. HBV could be transmitted through blood	110 (99.1)
9. HBV could be transmitted from a mother to his foetus	90 (81.1)
10. You are at a higher risk of HBV infection than the general population	87 (78.4)
11. There is a vaccine available against HBV	108 (97.3)
12. The minimum number of doses for a complete primary HBV vaccination is 3 doses	61 (55)
13. An immune response test should be done after HBV vaccination	38 (34.2)

HBV: hepatitis B virus.

### Awareness and perception of HBV vaccine, and vaccination status

One hundred and eight (97.3%) were aware of the existence of the HBV vaccine, but their knowledge of the vaccine was “poor” (44.6% of correct answers) (Table 1). Forty one (36.9%) participants considered the HBV vaccine completely safe.

Twenty (18%) participants were adequately vaccinated against HBV, 34 (30.6%) were inadequately vaccinated and 57 (51.4%) were not vaccinated. In addition, only 2 of the 20 (10%) adequately vaccinated participants had post-vaccination testing for antibodies against hepatitis B surface antigen, to confirm a good response and thus an effective protection against HBV infection. Among the 57 (51.4%) participants who had never had any dose of the HBV vaccine, the main reasons for not being vaccinated were lack of money to pay for the vaccine (45.6%), lack of sufficient information on the vaccine (17.5%), and lack of motivation (15.8%). Forty seven (42.3%) participants had been informed by their training institution about the importance of HBV vaccination. Participants who had been sensitized on the importance of HBV vaccination were more likely to be vaccinated than those who had not been sensitized (p < 0,001). Year IV medical students were less vaccinated than years V and VI (p < 0,001). Besides, there was no significant statistical association between HBV vaccination status and age (p = 0.758) or gender (p = 0.652) (Table 2).

**Table 2:** HBV vaccination status according to the demographic and academic characteristics among 111 medical students in Cameroon

**Characteristics**	**Not vaccinated**	**Inadequately vaccinated**	**Adequately vaccinated**	**p-value**
Age (mean ± SD)	23.1 ± 1.2	22.8 ± 1.0	23.3 ± 0.97	NS
Gender (% M / F)	50.9 / 49.1	47.1 / 52.9	60 / 40	NS
Time of the vaccination				
Before preclinical years	-	8	3	NS
Before clinical years	-	23	15	
During clinical years	-	3	2	
Academic level				
Year IV	15	2	1	< 0,001
Year V	9	28	11	
Year VI	33	4	8	
Sensitized on the importance of the HBV vaccine				
Yes	10	20	17	< 0,001
No	47	14	3	

NS: p-value is not significant.

## Discussion

This is the first survey on HBV vaccination among medical students in Cameroon. In this study we describe, among medical students in Cameroon, their knowledge of risk factors for HBV infection, history of accidental exposure to blood, awareness of HBV vaccine and their vaccination status. As part of occupational safety measures, all health care workers are required to be vaccinated against HBV [4,5]. Studies have reported highest occupational risk of HBV infection during health professional training [6]. Inadequate staff, lack of experience, insufficient training, duty overload and fatigue may lead to occupational sharp injuries [10,11]. We found that 55.9% of our participants had had at least one accidental exposure to blood since the beginning of their training, and up to 36 (58%) of them never reported these exposures. Comparatively, Okeke et al. in Nigeria found that 48% of medical students surveyed admitted having had a previous needlestick injury [12]. In a study among interns and medical students in Palestine, Al-Dabbas et al. reported that 40% of the study participants had experienced at least one needlestick injury, and failure to report the injury to health representatives was recorded for 48.6% of needlestick injuries [13]. Not reporting accidental exposure to blood increases the risk of HBV infection, since no post-exposure preventive measures are taken to reduce the risk of infection. Moreover, 39.3% of our participants had never considered the risk of HBV infection after exposure. Studies from Cameroon have reported that the prevalence of HBV infection among blood donors who were considered as apparently healthy adults was approximately 15% [14,15]. Considering the relative high HBV prevalence in the general population of Cameroon suggested by these studies and the fact that HBV have a 30% risk of contamination after exposure by a single needlestick injury in non-immune individuals [16], our findings show that medical students in Cameroon are at a high risk of HBV infection and highlight the necessity of HBV vaccination in this particular population.

Unfortunately, only 18% of our participants were adequately vaccinated. Studies have shown that awareness of risk among health care workers is an important factor affecting HBV vaccine uptake [17-19]. This low HBV vaccine uptake among our participants contrasts with their good knowledge of the risk factors of HBV infection, the fact that the majority (78.4%) considered that they are at a higher risk of HBV infection than the general population, their awareness of the existence of HBV vaccine and their willingness to recommend this vaccine to their classmates (87.4% of participants). This HBV vaccination uptake in our study population is considerably lower than those found in other studies. In Nigeria, Okeke et al., and Solofa et al. found a HBV vaccine coverage of 47.7% among medical students and 37.9% among dental students [12,20]. In Palestine, Al-Dabbas reported that 76.8% of medical students and 46.7% of interns in their study were vaccinated against HBV [12].

The main reasons reported by our participants for not being vaccinated were lack of time to attend vaccination (38.5%), lack of money to pay for the vaccine (23.1%) and lack of sufficient information on the vaccine (19.2%). Perception of incomplete safety of HBV vaccine by 49% of our participants could also explain the low vaccination rate reported in our study, since refusal of HBV vaccine has been found to be related to concern about vaccine side effects and fear of getting HBV infection from the vaccination [16]. We also found that only 42.3% of participants had been sensitized by the authorities of the faculty of medicine about the importance of HBV vaccination and that these students who had been sensitized where more adequately vaccinated (p < 0,001). Therefore, strongly recommending HBV vaccination and making the vaccine available free of charge should enhance vaccination uptake in medical trainees in Cameroon. Moreover, 21.7% (33/152) of the exposures in our participants had occurred within preclinical years during non-academic clinical traineeship. It would be good for medical students to be vaccinated when they enroll into the medical program.

Our study findings may be limited by the fact that we may have overestimated the proportion of the “adequately vaccinated” participants, because only 10% of these “adequately vaccinated” had an immune response test to confirm a good response of the HBV vaccine and thus an effective protection against HBV infection.

## Conclusion

Our study shows a high rate of accidental exposure to blood and very low HBV vaccination uptake in medical students in Cameroon, leading to a high occupational risk of HBV infection. HBV vaccination should be strongly recommended and the vaccine made available free of charge for medical students before the beginning of their training.

## Competing interests

The authors declare that they have no competing interests. They have not benefited from any sponsorship and funding.

## Authors’ contributions

JJNN designed the study, analyzed the data, drafted and revised the manuscript. JRNN contributed to the study design, collected and analyzed the data, and revised the manuscript. KKK collected the data and revised the manuscript. SNT and LAA revised the manuscript. All the authors revised and approved the final version of the manuscript.

## Pre-publication history

The pre-publication history for this paper can be accessed here:

http://www.biomedcentral.com/1472-6920/13/148/prepub
